# Nursing faculty academic incivility: perceptions of nursing students and faculty

**DOI:** 10.1186/s12909-017-1096-8

**Published:** 2017-12-13

**Authors:** Joshua K. Muliira, Jansi Natarajan, Jacoba van der Colff

**Affiliations:** 1 Department of Adult Health & Critical Care, College of Nursing, Sultan Qaboos University, P. O. Box 66 Al Khod, Muscat, Oman; 2 Department of Fundamentals & Administration, College of Nursing, Sultan Qaboos University, P. O. Box 66 Al Khod, Muscat, Oman; 3 Ministry of Health, Directorate of Nursing and Midwifery, Government of Sultanate of Oman, Muscat, Oman

**Keywords:** Nursing education, Safety, Professionalism, Incivility, Undergraduate

## Abstract

**Background:**

Incivility in nursing education can adversely affect the academic environment, the learning outcomes, and safety. Nursing faculty (NF) and nursing students (NS) contribute to the academic incivility. Little is known about the extent of NF academic incivility in the Middle East region. This study aimed at exploring the perceptions and extent of NF academic incivility in an undergraduate nursing program of a public university in Oman.

**Methods:**

A cross sectional survey was used to collect data from 155 undergraduate NS and 40 NF about faculty academic incivility. Data was collected using the Incivility in Nursing Education Survey.

**Results:**

The majority of NS and NF had similar perceptions about disruptive faculty behaviors. The incidence of faculty incivility was low (Mean = 1.5). The disruptive behaviors with the highest incidence were arriving late for scheduled activities, leaving schedule activities early, cancelling scheduled activities without warning, ineffective teaching styles and methods, and subjective grading. The most common uncivil faculty behaviors reported by participants were general taunts or disrespect to other NF, challenges to other faculty knowledge or credibility, and general taunts or disrespect to NS.

**Conclusion:**

The relatively low level of NF academic incivility could still affect the performance of some students, faculty, and program outcomes. Academic institutions need to ensure a policy of zero tolerance to all academic incivility, and regular monitoring and evaluation as part of the prevention strategies.

## Background

Incivility is one the major challenges facing nursing education and learning processes in the classroom, clinical, and online for distance education programs [1–3]. Incivility in nursing education has been defined as rude or disruptive behaviors which often result into psychological or physiological distress for the people involved [4]. In the academic setting incivility can be any action that impedes the development of a harmonious and cooperative learning atmosphere [5], and can be initiated by either the nursing students (NS) or nursing faculty (NF). Regardless of the source, incivility in nursing education undermines the culture of safety [5-7], and jeopardizes welfare and goal attainment by all the stakeholders (NS, NF, other staff members, and patients).

The majority of studies about incivility (in nursing education) have focused on NS and have been conducted in North America [8, 9]. This paper focuses on incivility stemming from NF in Oman (a country in the Middle East). This focus is needed because to comprehensively prevent academic incivility, one has to understand all its sources and forms. In all nursing programs (regardless of the setting), NF members are expected to be role models and mentors of future nursing professionals. However, increasingly NF members are curtailing their efforts towards role modeling of future nurses with incivility acts in the academic setting. Studies conducted in USA show that in some nursing programs up to 35% of NS experience at least one NF who puts them down or is condescending during educational experiences [10]. There are no studies from other parts of the world that can be used for comparison.

The other forms of NF incivility reported by NS include subjective evaluation, targeting and weeding out practices [11]. The targeting practices are mostly prevalent during clinical learning and clinical evaluation, and end up hindering students’ learning, self-esteem, self-efficacy, confidence, and professional formation [11]. Academic incivility is also evident in reports which show that NF pressurize students to conform to unreasonable academic demands [12]. For instance in the clinical or laboratory setting, some NF ask students to perform a skill on the first or second day of starting the learning experience and expect them to perform without a mistake. When the students make a mistake they may be told from day one that “you have failed” or “you will not perform well in the course”. Such actions by the NF cause the NS to feel pressurized to learn instantly in every experience.

NF academic incivility also leads to increased stress and anxiety among NS, and this in-turn propagates an increase in student academic incivility [13]. In the learning environment when NS are stressed as a result of lack of support, unequal treatment, and other unprofessional behaviors of the NF, they may lose control of their emotions, and become helpless and frustrated. The outcomes of NS feelings of helplessness and frustration affect their interactions with the NF, and these student emotions are portrayed as disrespect to NF, side talks in class, failure to control what is said to NF, and others [13]. The other outcomes of NF initiated academic incivility include student avoidance of the faculty, feelings of nervousness, anxiety, depression, and low satisfaction with the nursing program among NS [10]. During classroom learning activities a dissatisfied and depressed NS may be perceived by the NF as disinterested or uncivil.

Academic incivility has the potential of terminating the learning process because it diminishes the trust in NF leadership and it decreases NS productivity [14]. Therefore, academic incivility initiated by NF at the least impedes the learning process, and can lead to physical and emotional disengagement, and increases the potential for reciprocation [15]. Hence, the recommendations calling for implementation of polices and strategies that ensure continuous monitoring of potential sources of incivility in nursing education [16].

Apart from NS, the other stakeholders impacted by NF incivility are the other faculty and staff members, and patients in the academic or clinical settings. Incivility generates a culture of intimidation and this leads to an environment of hostility, disrespect, low morale, high staff turnover, distraction, errors, and diminished patient safety [17]. This negative impact of incivility has also been highlighted by the office of quality and safety of the Joint Commission, a hospital accrediting body [18]. The Joint Commission articulated that intimidating behaviors, disrespectful behaviors, use of inappropriate words, shaming, and unjustified comments in the clinical or work environments disrupts the culture of safety [18]. A study of novice NF found that faculty to faculty incivility led them to feel a sense of rejection, a need to employ specific behaviors to cope with uncivil colleagues, a need to quit the faculty position, a perception that others wanted them to fail, a perception that senior faculty were territorial and possessive, and that no one was available to help [19]. This wide reach of incivility informs us that all nursing institutions need to be reliably aware of the state of academic incivility.

Currently in the nursing program where this study was conducted, the majority of NF members are foreigners and have different cultures from that of their NS. This is a potential ingredient for cultural differences and misunderstandings in a rigorous academically demanding nursing program [2]. The need for prevention and early detection of academic incivility, led us to conduct a deliberate study to document the NF and NS academic incivility. Literature shows that some incivility at the workplace is due to the perpetrators’ unintentional violation of social norms or unconscious prejudice [20]. A study focusing on incivility and organization diversity also revealed that individuals are more likely to experience civility in the hospital setting, if the hospital ethnic diversity or demography is closely representative of the community [21].

Therefore the main purpose of the study was to explore the perceptions and extent of NF and NS academic incivility in an undergraduate nursing program based in Oman. The findings about NS academic incivility have been reported elsewhere [22]. Since there were no studies which have addressed NF academic incivility from the Middle East region, this paper addresses this gap using the perspectives of NF and NS.

## Methods

Data was collected from a cross section of NS and NF in a bachelor of science in nursing (BSN) program of a public university in Oman. The program has approximately 58 NF and enrolls 100 direct entry students and 20 bridging students annually in the 4 year and 2 year BSN program, respectively. The NS come from different regions of Oman and the NF (not more than 7 from each country) are from a variety countries such as USA, Jordan, Uganda, Philippines, India and others.

### Sample

The participants were the NF and NS in the BSN program. A convenience sampling approach was used to recruit the participants. The NS were included if they met the criteria of: enrolled in the BSN program; at least 18 years of age; spent at least one year in the BSN program, officially registered in the current semester; and completed at least one clinical course (clinical learning exposure). The NF were included if they met the criteria of: worked at the college for at least 12 months; has responsibilities which require regular teaching and direct contact with undergraduate students. The NF who were involved in only administrative responsibilities were excluded from the sample. A total of 50 NF and 200 NS were approached and invited to participate in the study. The response rate for the NF and NS was 80% and 78%, respectively.

### Study instrument

A questionnaire written in English was used to collect data. English is the language of instruction in the BSN program. The questionnaire was comprised of four sections (demographic characteristics and the incivility in nursing education survey). The NF academic incivility was measured with the incivility in nursing education (INE) survey. The INE survey was designed to measure NS and NF perceptions and experiences of academic incivility [23]. The INE survey contains items focusing on NS and NF disruptive and threatening behaviors [23]. There were no modifications made to the INE. This paper presents the quantitative findings about NF academic incivility.

The participants were first required to indicate whether a NF behavior is disruptive with a response of “yes” and “no”. And then on the same item the participants were required to rate how often they have experienced or seen the behavior in the academic environment in the past 12 months using a 4-point Likert scale (Always = 4; Usually = 3; Sometimes = 2; and Never =1). The 4-point Likert scale is used to determine the frequency of specific acts of NF academic incivility observed or experienced by participants. In other studies the Cronbach’s alpha was found to be between 0.84 to 0.88 for the faculty behavior items of the INE survey [23, 24]. In the present study, the faculty behavior items of the INE survey had a Cronbach’s alpha of 0.80.

### Ethical considerations

The study was approved by the research and ethics committee of the College of Nursing. All the participants were given clear explanations of the study purpose, procedures, and risks. The participants were required to read and sign the informed consent form before completing the study questionnaire. The participants were informed of their right to stop participation in the study at any time without any penalty. The data for this study was only accessed by the investigators and did not include any participant identifying information.

### Data collection procedure

After approval of the study by the research and ethics committee of the College of Nursing, the study was advertised to both the NF and NS through the college website, emails and fliers. The data for this study was collected from March to December, 2016. The study was given permission to access the college website to get a list of all NF. The NF were approached while in their offices. The research assistant (RA) made appointments to come back later to collect data from NF who were busy or unavailable after the first attempted contact. The NS were approached to collect data at the end of scheduled classes. The class schedules posted on the college website were used to plan the data collection dates. The participants had to read and sign the informed consent form. After completing the consent process the study questionnaire was administered in a private space for NS and in the office for the NF.

### Data analysis

The data was analyzed using Statistical Package for Social Science (SPSS Version 22.0). Data was checked for normality with the Shapiro-Wilk statistic and was normally distributed (W = 0.934; *p* = 0.325). Descriptive statistics were used to summarize the participants’ perceptions of NF incivility and the common uncivil faculty behaviors. The independent samples t-test and Chi-square test were used to evaluate the differences between NS and NF perceptions of NF academic incivility. The level of significance for all analyses was set at ≤0.05 (two tailed).

## Results

### Characteristics of the participants

The results presented in the Table 1 show that majority of faculty were above 30 years of age (70%), had a masters or doctorate degree (75%) and were teaching courses in the third and fourth level (81%) of the BSN program. The average years spent as nurse educators among the faculty was 11.6 years. The majority of NS reported a grade point average above 2.0 out of 4 (97.5%). The NS were familiar with the behaviors expected of health care professionals since a large number reported having a family member who is a nurse (50.3%) or a doctor (30.3%).

**Table 1:** Characteristics of nursing students and faculty

Participants	Characteristic	Category	Frequency (%)
Nursing faculty (*n* = 40)	Age in years (Mean = 36.16, SD = 9.77)	18–21	3 (7)
		22–30	10 (23.2)
		> 30	30 (69.8)
	Gender	Male	9 (28.1)
		Female	23 (71.9)
	Academic Qualification	Bachelors	6 (15)
		Masters	24 (60)
		Doctorate	10 (25)
	Number of years as a nurse educator (Mean = 11.56; SD = 8.34)	< 5	8 (22.2)
		6–15	21 (58.3)
		16–25	4 (11.2)
		26–30	3 (8.3)
	Average number of students in a theory class (Mean = 35.31; SD = 20.629)	< 20	7 (17.1)
		21–40	10 (24.4)
		41–60	8 (19.5)
		> 60	16 (39)
	Level of nursing courses in the BSN Program commonly taught	Third level	10 (37)
		Fourth level	12 (44.4)
		All levels	5 (18.5)
Nursing students (*n* = 155)	Age in years (Mean = 22.19; SD = 3.59)	18–21	118 (71.1)
		22–30	32 (19.3)
		>31	16 (9.6)
	Gender	Male	43 (33.3)
		Female	86 (66.7)
	Cumulative grade point average (self-reported)	0–2	4 (2.5)
		2.1–4	158 (97.5)
	Year of study in the BSN program	Two	23 (18.4)
		Three	69 (55.2)
		Four	33 (26.4)
	Has a family member who is a nurse	Yes	77 (50.3)
		No	76 (49.7)
	Has a family member who is a doctor or any other health profession	Yes	47 (30.3)
		No	108 (69.7)

### Faculty and student perception of disruptive faculty behaviors

The results about NS and faculty perceptions of disruptive NF behaviors are summarized in Table 2. The majority of faculty members (≥50%) felt and considered the listed NF behaviors to be disruptive. Similarly, 50% or more of all the NS perceived the faculty behaviors listed in Table 2 to be disruptive, except leaving scheduled activities early (44.7%); refusing or being reluctant to answer questions (49.7%); and threatening to fail a student for not complying with faculty demands (43.8%).

**Table 2:** Nursing student and faculty perceptions of disruptive faculty behaviors

Nursing faculty behavior	Response	Faculty (*n* = 40) F (%)	Student (*n* = 155) F (%)	Chi Square (Ӽ^2^) and *p*-value
Arriving late for scheduled activities	Yes	30(78.9)	100(66.7)	Ӽ^2^ = 2.143 *p* = 0.143
	No	8(21.1)	50(33.3)	
Leaving scheduled activities early	Yes	27(71.1)	67(44.7)	Ӽ^2^ = 8.444 p = 0.004
	No	11(28.9)	83(55.3)	
Cancelling scheduled activities without warning	Yes	31(81.6)	95(64.6)	Ӽ^2^ = 3.995 p = 0.046
	No	7(18.4)	52(35.4)	
Being unprepared for scheduled activities	Yes	25(65.8)	92(62.2)	Ӽ^2^ = 0.170 *P* = 0.680
	No	13(34.2)	56(37.8)	
Not allowing open discussion	Yes	21(58.3)	77(52)	Ӽ^2^ = 0.463 *p* = 0.496
	No	15(41.7)	71(48)	
Refusing to allow make up exams, extensions, or grade changes	Yes	18(50)	93(63.7)	Ӽ^2^ = 2.278 *p* = 0.131
	No	18(50)	53(36.3)	
Ineffective teaching style/methods	Yes	26(72.2)	109(73.2)	Ӽ^2^ = 0.013 *p* = 0.910
	No	10(27.8)	40(26.8)	
Deviating from the course syllabus, changing assignments or test dates	Yes	22(61.1)	89(61.4)	Ӽ^2^ = 0.001 *p* = 0.976
	No	14(38.9)	56(38.6)	
Being inflexible, rigid and authoritarian	Yes	26(70.3)	96(65.8)	Ӽ^2^ = 0.271 *p* = 0.603
	No	11(29.7)	50(34.2)	
Punishing the entire class for one student’s misbehavior	Yes	19(52.8)	98(66.7)	Ӽ^2^ = 2.419 *p* = 0.120
	No	17(47.2)	49(33.3)	
Making statements about being disinterested in the subject matter	Yes	22(59.5)	88(60.3)	Ӽ^2^ = 0.008 *p* = 0.928
	No	15(40.5)	58(39.7)	
Being distant and cold towards others (unapproachable reject students opinions)	Yes	19(51.4)	89(61)	Ӽ^2^ = 1.127 *p* = 0.289
	No	18(48.6)	57(39)	
Refusing or reluctant to answer questions	Yes	18(50)	73(49.7)	Ӽ^2^ = 0.001 *p* = 0.971
	No	18(50)	74(50.3)	
Subjective grading	Yes	27(73)	82(61.2)	Ӽ^2^ = 1.741 *p* = 0.187
	No	10(27)	52(38.8)	
Making condescending remarks or put downs	Yes	23(65.7)	83(58.5)	Ӽ^2^ = 0.617 *p* = 0.432
	No	12(34.3)	59(41.5)	
Exerting superiority rank over others	Yes	26(72.2)	75(54.7)	Ӽ^2^ = 3.584 *p* = 0.058
	No	10(27.8)	62(45.3)	
Threatening to fail a student for not complying with faculty’s demands	Yes	18(51.4)	64(43.8)	Ӽ^2^ = 0.256 *p* = 0.613
	No	17(48.6)	82(56.2)	
Making rude gestures or behaviors towards others	Yes	21(60)	77(52.4)	Ӽ^2^ = 0.660 *p* = 0.416
	No	14(40)	70(47.6)	
Ignoring disruptive student behavior	Yes	24(66.7)	75(51.4)	Ӽ^2^ = 2.724 *p* = 0.099
	No	12(33.3)	71(48.6)	
Being unavailable outside of class (not returning calls or emails, not maintaining office hours)	Yes	23(63.9)	88(59.5)	Ӽ^2^ = 0.237 *p* = 0.626
	No	13(36.1)	60(40.5)	

More NF (71.1%) perceived the faculty behaviors of leaving scheduled activities early as being disruptive compared to NS (44.7%). This difference was significant, Ӽ^2^ = (1, *N* = 195) = 8.44, *p* = 0.004. Also more NF (81.6%) perceived the faculty behavior of canceling scheduled activities without warning as being disruptive compared to NS (64.6%). This difference was also significant, Ӽ^2^ = (1, N = 195) = 3.99, *p* = 0.046. These findings reveal the expected sense of accountability among NF and a tendency in NS of preferring to do less (ending schedules activities early or canceling of scheduled activities). One of the goals of nursing education is to impart professional accountability into NS and the students perceptions showed above highlight the need for more emphasis on professional accountability.

### Nursing student and faculty experiences with faculty incivility

The results presented in Table 3 show that the most commonly experienced uncivil NF behaviors by faculty were general taunts or disrespect to other faculty (43.2%), challenges to other faculty knowledge or credibility (28.9%), and general taunts or disrespect to NS (23.1%). The uncivil NF behaviors commonly experienced by NS were general taunts or disrespect to students (29.1%), general taunts or disrespect to other NF (17%), and challenges to other faculty knowledge or credibility (34.9%).

**Table 3:** Student and faculty experience with uncivil faculty behaviors

Faculty behavior	Response	Faculty (n = 40) F (%)	Student (n = 155) F (%)	Chi Square (Ӽ^2^), *p*-value
General taunts or disrespect to other students	Yes	9(23.1)	44(29.1)	Ӽ^2^ = 0.566 *p* = 0.452
	No	30(76.9)	107(70.9)	
General taunts or disrespect to other faculty	Yes	16(43.2)	25(17)	Ӽ^2^ = 11.750 p = 0.001
	No	21(56.8)	122(83)	
Challenges to other faculty knowledge or credibility	Yes	11(28.9)	51(34.9)	Ӽ^2^ = 0.483 *p* = 0.487
	No	27(71.1)	95(65.1)	
Harassing comments (racial, ethnic, gender) directed at students	Yes	5(13.2)	25(16.9)	Ӽ^2^ = 0.312 *p* = 0.577
	No	33(86.8)	123(83.1)	
Harassing comments (racial, ethnic, gender) directed at other faculty	Yes	8(21.6)	20(14.2)	Ӽ^2^ = 1.223 *p* = 0.269
	No	29(78.4)	121(85.8)	
Vulgarity directed at students	Yes	7(18.4)	18(13.3)	Ӽ^2^ = 0.621 *p* = 0.431
	No	31(81.6)	117(86.7)	
Vulgarity directed at other faculty	Yes	8(21.1)	16(11.9)	Ӽ^2^ = 2.047 *p* = 0.152
	No	30(78.9)	118(88.1)	
Inappropriate emails to other students	Yes	2(5.4)	8(5.4)	Ӽ^2^ = 0.000 *p* = 0.993
	No	35(94.6)	141(94.6)	
Inappropriate emails to faculty	Yes	3(8.1)	15(10.3)	Ӽ^2^ = 0.165 *p* = 0.684
	No	34(91.9)	130(89.7)	
Threats of physical harm against other students	Yes	2(5.3)	13(8.7)	Ӽ^2^ = 0.492 *p* = 0.483
	No	36(94.7)	136(91.3)	
Threats of physical harm against other faculty	Yes	3(7.9)	11(7.7)	Ӽ^2^ = 0.002 *p* = 0.967
	No	35(92.1)	132(92.3)	
Property damage	Yes	5(13.2)	18(12.6)	Ӽ^2^ = 0.009 *p* = 0.925
	No	33(86.8)	125(87.4)	
Statements about having access to weapons	Yes	2(5.3)	11(4.8)	Ӽ^2^ = 0.246 *p* = 0.620
	No	36(94.7)	134(92.4)	

More NF (43.2%) had experienced faculty behavior of general taunts or disrespect to other faculty compared to NS (17%). This difference was significant, Ӽ^2^ = (1, *N* = 195) = 11.75, *p* = 0.001. The limited NS experience of faculty behavior of general taunts or disrespect to other faculty may be because faculty to faculty uncivil incidences take place in spaces not shared with students (faculty offices, meeting rooms and others of this nature). The percentage of NF (43.2%) who experienced general taunts or disrespect to other faculty is large and this indicates an area of improvement.

### Incidence of disruptive faculty behaviors

The NS and NF were asked to rate how often they have experienced or seen each faculty behavior in the past 12 months using a 4 point Likert scale. The NF behaviors with a mean score of 2 and above were considered to be significant NF academic incivility. The average rating by both the NS and NF of all the 20 disruptive behaviors was 1.5 (low), showing that they were collectively occurring less than sometimes. The results presented in Table 4 show that the NF behaviors with the highest incidence were: arriving late for scheduled activities; leaving scheduled activities early; canceling scheduled activities without warning; ineffective teaching styles and methods; and subjective grading.

**Table 4:** Incidence of uncivil faculty behaviors based on experiences students and faculty

Faculty behavior	Rater	N	M	SD	SEM	t	df	*p*
Arriving late for scheduled activities	Faculty	31	1.806	0.402	0.07	1.14	147	0.26
	Student	118	1.703	0.459	0.04			
Leaving scheduled activities early	Faculty	31	1.742	0.445	0.08	2.97	147	0.00
	Student	118	1.449	0.500	0.05			
Cancelling scheduled activities without warning	Faculty	31	1.839	0.374	0.07	1.76	147	0.05
	Student	118	1.678	0.469	0.04			
Being unprepared for scheduled activities	Faculty	31	1.645	0.486	0.09	0.09	147	0.92
	Student	118	1.636	0.483	0.04			
Not allowing open discussion	Faculty	31	1.581	0.502	0.09	0.46	147	0.65
	Student	118	1.534	0.501	0.05			
Refusing to allow make up exams, extensions, or grade changes	Faculty	31	1.548	0.506	0.09	−0.71	147	0.48
	Student	118	1.619	0.488	0.05			
Ineffective teaching style/methods	Faculty	31	1.710	0.461	0.08	−0.40	147	0.69
	Student	118	1.746	0.437	0.04			
Deviating from the course syllabus, changing assignments or test dates	Faculty	31	1.613	0.495	0.09	−0.41	147	0.68
	Student	118	1.653	0.478	0.04			
Being inflexible, rigid and authoritarian	Faculty	31	1.677	0.475	0.09	−0.09	147	0.92
	Student	118	1.686	0.466	0.04			
Punishing the entire class for one student’s misbehavior	Faculty	31	1.516	0.508	0.09	−1.49	147	0.14
	Student	118	1.661	0.475	0.04			
Making statements about being disinterested in the subject matter	Faculty	31	1.581	0.502	0.09	−0.47	147	0.64
	Student	118	1.627	0.486	0.05			
Being distant and cold towards others (unapproachable reject students opinions)	Faculty	31	1.516	0.508	0.09	−0.94	147	0.35
	Student	118	1.610	0.490	0.05			
Refusing or reluctant to answer questions	Faculty	31	1.548	0.506	0.09	0.39	147	0.70
	Student	118	1.509	0.502	0.05			
Subjective grading	Faculty	31	1.742	0.445	0.08	1.44	147	0.15
	Student	118	1.602	0.492	0.05			
Making condescending remarks or put downs	Faculty	31	1.645	0.486	0.09	0.35	147	0.72
	Student	118	1.610	0.490	0.05			
Exerting superiority rank over others	Faculty	31	1.677	0.475	0.09	1.27	147	0.21
	Student	118	1.551	0.500	0.05			
Threatening to fail a student for not complying with faculty’s demands	Faculty	31	1.516	0.508	0.09	−0.60	147	0.55
	Student	118	1.576	0.496	0.05			
Making rude gestures or behaviors towards others	Faculty	31	1.613	0.495	0.09	0.78	147	0.44
	Student	118	1.534	0.501	0.05			
Ignoring disruptive student behavior	Faculty	31	1.677	0.475	0.09	1.69	147	0.09
	Student	118	1.509	0.502	0.05			
Being unavailable outside of class (not returning calls or emails, not maintaining office hours)	Faculty	31	1.645	0.486	0.09	0.44	147	0.66
	Student	118	1.602	0.492	0.05			

*M* Mean, *SD* Standard Deviation, *SEM* Standard Error Mean, *t* t-test statistic, *df* degrees of freedom, *p* p-value

The faculty behavior of leaving scheduled activities early was reported to occur more often by NF (mean = 1.74 ± 0.45) as compared to NS (mean = 1.45 ± 0.50). This difference was significant, t(147) = 2.97, *p* = 0.00. The faculty behavior of canceling scheduled activities without warning was also reported to occur more often by NF (mean = 1.84 ± 0.37) compared to NS (mean = 1.68 ± 0.47). This difference was also significant, t(147) = 1.76, *p* = 0.05. Being available to teach during scheduled activities is one of the primary responsibilities of NF, and to show accountability NF must attend such activities without missing. Therefore any slight change in schedule or canceling is most likely to be viewed by other NF as lack of accountability or not doing your job. The NS on the other hand may not take the act of ending scheduled activities early or canceling scheduled activities seriously, because they view it as an opportunity to do less or rest.

## Discussion

To our knowledge this the first study to explore the phenomenon of NF academic incivility in any institution in the Middle East region. The findings show that both the NS and faculty reported a low incidence of NF initiated academic incivility. However even this little can affect a certain section of the NS, NF, the learning process, and has to be promptly addressed. Prompt intervention to ameliorate any kind of academic incivility is essential, because incivility can lead to a weakened learning environment, reduced safety, poor workforce behaviors, and violence [3].

In nursing education the NF are expected to be the role models of caring and respect in the formal and hidden curriculum, and this in turn contributes to the positive formation of future nurses [11]. But when the NF demonstrate any behavior which is considered disruptive or uncivil they fail in their responsibility of; grooming future professional nurses; ensuring a safe and effective academic environment; and promoting patient safety. In the current study the majority (≥50%) of NS and NF agreed on what is considered disruptive NF behaviors. This informs us that the behaviors of NF which are uncivil are easy to identify and cannot be mistaken for personal idiosyncrasies or ones way of doing things. Attempts to excuse NF academic incivility as personal style or ways of doing things are at the least another act of incivility.

The findings show that the most commonly experienced uncivil faculty behaviors by NF were general taunts or disrespect to other faculty (43.2%), challenges to other faculty knowledge or credibility (28.9%), and general taunts or disrespect to students (23.1%). The uncivil faculty behaviors commonly experienced by NS were general taunts or disrespect to students (29.1%), general taunts or disrespect to other faculty (17%), and challenges to other faculty knowledge or credibility (34.9%). In a study conducted in a nursing institution located in USA, the NF and NS reported slightly higher incidences of challenges to other faculty’s knowledge or credibility (43.5%) and general taunts or disrespect to students (25.3%) [25].

The findings of the current study led us to estimate that at least 43% of NF and 35% of NS experienced an act of NF incivility. Compared to other studies this incidence of NF incivility is low. In one study conducted in USA more than 50% of NS in the BSN program experienced an act NF incivility [10], while in another, 61% of the NS reported uncivil or humiliating behaviors by NF [12]. An earlier study showed that 88% of the NS reported experiencing at least one instance of uncivil NF behaviors [26]. However it should be noted that these studies used a different measure of NF incivility and were conducted in settings where freedom of speech and expression are valued.

Literature shows that incivility at the workplace may arise from the perpetrators’ unintentional violation of social norms and unconscious prejudice [20]. Due to the diverse backgrounds of the NF in the college where the study was done, we expected the incidence of NF incivility to be high, but it was low. The low incidence of NF incivility could be due to the lack of a dominant group among the NF. The NF from the same nation or cultural background were at most seven, but all the NS are from the same cultural, nationand religious background (Muslim and Omani). This suggests that the NS are demographically the dominant group and therefore more likely to be the perpetrators and not the victims of incivility.

The large majority of NF are expatriates and experienced nurse educators (mean years of experience = 11.56 years) from other countries. It seems in their work, the NF try to be professional and careful to ensure NS satisfaction. The expatriate NF also do not have tenure at the University and their work contracts get renewed every 2 to 3 years based on performance in teaching, research and community service. The study was also carried out in a Muslin country where religion is a central part of every activity in the day to day lives of NS and nationals. The religious norms, expectations and influence on the NS and NF may have caused a tendency of forgiveness and minimizing uncivil behaviors. However as the number of Omani NF increases in nursing education, there will be a need for additional studies in future to evaluate the changes in the incidence of NF incivility.

In the current study, the NF behaviors with the highest incidence were; arriving late for scheduled activities; leaving scheduled activities early; canceling scheduled activities without warning; ineffective teaching styles and methods; and subjective grading. Similarly, a phenomenological study of NS focusing on NF uncivil behavior reported that subjective evaluation and weeding out or targeting practices were a common and distressful practice among NF [11]. In another study 54% of the NS reported threatening of low grades as one of the common uncivil NF behaviors [12].

The NF behaviors with the highest incidence in our study were mostly related to NF professionalism and commitment to teaching and learning activities. In other studies the NS reported uncivil communication patterns as the most commonly occurring example of uncivil behaviors [10]. The examples of uncivil communications patterns include belittling, yelling, and talking to other students about the performance of another student [12]. This difference in perception between nursing students from the Middle East and North America may be due to cultural and societal differences. There were no quantitative studies reporting about faculty experiences and perceptions that could be used for comparison.

The findings of our study show that NF academic incivility was low, and that nursing education in Oman is not immune to academic incivility. This realization should increase the propensity of nursing institutions towards proactive monitoring and preventive strategies to eliminate academic incivility. Some of the needed actions include zero tolerance policies, providing faculty with the needed support [3], faculty development and mentoring activities focusing on civility, and regular monitoring and evaluation of the state of academic civility. Preventive actions which are beyond the normal academic rules and regulations are needed because of the potential outcomes of any level of NF academic incivility. It has been reported elsewhere that NF academic incivility is associated with program dissatisfaction and negative student psychological outcomes (anxiety, depression and nervousness) [26].

### Implications for nursing education

In nursing education, the public and students want their NF to maintain a conducive academic environment and to set good examples of civility [13]. The NF are also expected to demonstrate professionalism during all relationships and interactions with NS and others involved in the teaching learning process. Any tendency towards incivility undermines the learning process, nursing ethics, well-being, and the profession at large. The outcomes of any level of NF incivility have a negative impact that the profession cannot afford to bear. Hence, nursing education institutions need to ensure zero tolerance policies and preventive strategies that focus on elimination of incivility.

Contemporary nursing education requires continuous engagement in quality improvement processes such as accreditation in order to maintain the standards of professional education. Similar approaches are needed to address incivility. There is need for continuous monitoring and evaluation of the state of incivility in nursing education. Additionally, there is need for studies focusing on interventions, policies, and other modalities which are effective at preventing or curtailing academic incivility. Such strategies will provide the needed tools that can be adopted and tailored to the diverse academic settings where nursing education is provided.

### Limitations

The findings of the study should be considered in view of its limitations. Although the response rate for the NF and NS was 80% and 78%, respectively, the data was collected from one nursing program in Oman. The data was collected using self-report method. The study used the original version of the Incivility in Nursing Education survey (INE) and not the most recent revised version (INE-R) [27]. The INE has not been previously used in the Oman or the Middle East region. The questionnaire was pre-tested and found to be clear and understandable by the participants. The NF and NS could have been exposed to higher levels of incivility in other settings such as the hospital and this could have led them to view their experiences with NF as normal. The study was limited by lack of literature about incivility in nursing education specific to the Middle East region.

## Conclusion

Regardless of the country, in nursing education the teaching-learning process involves several stakeholders (NF, NS, patients and others), and during this process the NF and NS experience real challenging behaviors some of which can amount to incivility. Incivility in nursing education can happen anywhere and it impacts all stakeholders. The NF have a greater moral, ethical and professional responsibility to avoid the perpetuation of incivility because it undermines the teaching–learning process, the culture of safety, and efforts to role-model future nurses.

## Abbreviations

BSN: Bachelor of Science in Nursing
INE: Incivility in nursing education
NF: Nursing faculty
NS: Nursing students
RA: Research assistants
USA: United States of America
